# Human Brain Lipidomics: Investigation of Formalin Fixed Brains

**DOI:** 10.3389/fnmol.2022.835628

**Published:** 2022-06-16

**Authors:** Aaron W. Beger, Kathleen A. Hauther, Beatrix Dudzik, Randall L. Woltjer, Paul L. Wood

**Affiliations:** ^1^Department of Anatomy, DeBusk College of Osteopathic Medicine, Lincoln Memorial University, Harrogate, TN, United States; ^2^Metabolomics Unit, College of Veterinary Medicine, Lincoln Memorial University, Harrogate, TN, United States; ^3^Department of Neurology, Oregon Health Science University, Portland, OR, United States; ^4^Portland VA Medical Center, Portland, OR, United States

**Keywords:** diacylglycerol, triacylglycerol, hexosyl ceramide, cerebroside, glucosylceramide, galactosylceramide

## Abstract

Human brain lipidomics have elucidated structural lipids and lipid signal transduction pathways in neurologic diseases. Such studies have traditionally sourced tissue exclusively from brain bank biorepositories, however, limited inventories signal that these facilities may not be able to keep pace with this growing research domain. Formalin fixed, whole body donors willed to academic institutions offer a potential supplemental tissue source, the lipid profiles of which have yet to be described. To determine the potential of these subjects in lipid analysis, the lipid levels of fresh and fixed frontal cortical gray matter of human donors were compared using high resolution electrospray ionization mass spectrometry. Results revealed commensurate levels of specific triacylglycerols, diacylglycerols, hexosyl ceramides, and hydroxy hexosyl ceramides. Baseline levels of these lipid families in human fixed tissue were identified *via* a broader survey study covering six brain regions: cerebellar gray matter, superior cerebellar peduncle, gray and subcortical white matter of the precentral gyrus, periventricular white matter, and internal capsule. Whole body donors may therefore serve as supplemental tissue sources for lipid analysis in a variety of clinical contexts, including Parkinson’s disease, Alzheimer’s disease, Lewy body dementia, multiple sclerosis, and Gaucher’s disease.

## Introduction

Lipidomic investigations of the human brain have worked to elucidate the biochemical underpinnings of neurodegenerative (Xicoy et al., 2019; Kao et al., 2020) and psychiatric (Walther et al., 2018; Wood, 2019) diseases. Such studies have traditionally sourced tissue exclusively from brain bank biorepositories. However, declining autopsy rates (Burton and Underwood, 2007) have led to waning inventories (Kretzschmar, 2009), limiting sample sizes, and signaling that these facilities may be unable to meet the demands of this emerging field. Brain banks also incur significant staffing and equipment costs (Hulette, 2003), further restraining their growth. Seeking alternative tissue sources could reduce the burden on biorepositories while facilitating the expansion of human brain lipidomics.

To address this, we compared the lipid concentrations of fresh human brain tissue samples with those of formalin fixed brains of whole body donors willed to our institution, with the aim of introducing these subjects as a possible supplemental tissue source for lipid analysis. As formalin fixation is common practice for histopathological studies and long-term storage of tissue, its influence on the cerebral lipidome has been reported, albeit limitedly. 4% formaldehyde rapidly degrades phosphatidylethanolamines (Davison and Wajda, 1962; Gaudin et al., 2014) possibly due to modification of covalent bonds in their polar head groups (Gaudin et al., 2014). Meanwhile, 10% non-buffered formaldehyde was found to have minimal influence on phosphatidylcholine, sphingomyelin and gangliosides of rat brains and human cortical gray and subcortical white matter (Carter et al., 2011; Harris et al., 2020) with stability of sphingomyelin having been demonstrated in the human corpus callosum up to 24 years post-fixation (Heslinga and Deierkauf, 1962). While these findings help inform the utility of formalin fixed brains in lipidomics, the issue of limited source tissue persists.

Here we report the lipid families that were found to be insignificantly altered between fresh and fixed human cortical gray matter of the frontal lobe. High resolution electrospray ionization mass spectrometry revealed commensurate levels of specific diacylglycerols, triacylglycerols, hexosyl ceramides, and hydroxy hexosyl ceramides. An additional survey study provided baseline levels of these lipid families in six additional regions of fixed brain tissue: cerebellar cortical gray matter, white matter of the superior cerebellar peduncle, cortical gray and subcortical white matter of the precentral gyrus (primary motor cortex), periventricular white matter, and white matter of the posterior limb of the internal capsule.

## Results

### Comparative Analysis

**FIGURE 1:**
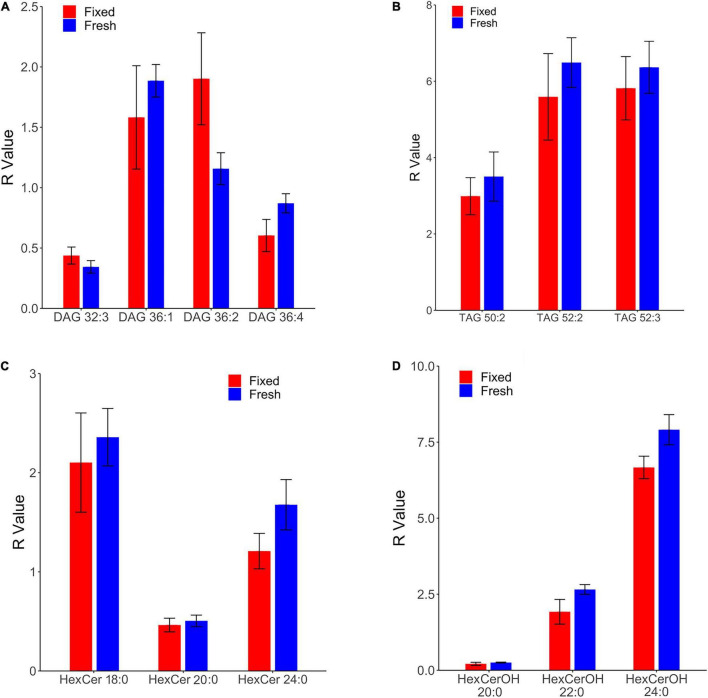
Comparison of lipid concentrations in fresh and fixed samples of human frontal cortical gray matter reveals commensurate levels of four lipid families. *R* values of fresh and fixed samples were found to differ insignificantly using two-sample *t*-tests (*p* > 0.05). Relative levels were calculated as a ratio of the peak area of the endogenous lipid to the peak area of the assigned internal standard, and were subsequently corrected for the wet weight of the sample to obtain the *R* value (relative level*(10/wet weight[mg])). **(A)** Four species of diacylglycerol (DAG) were found in commensurate levels in fresh and fixed brain tissue. **(B)** Three species of triacylglycerol (TAG) were found in commensurate levels in fresh and fixed brain tissue. **(C)** Three species of hexosyl ceramide (HexCer) were found in commensurate levels in fresh and fixed brain tissue. **(D)** Three species of hydroxy hexosyl ceramide (HexCerOH) were found in commensurate levels in fresh and fixed brain tissue.

Comparison of fixed and fresh human frontal cortical gray matter revealed commensurate relative levels in 13 lipid species that collectively belong to four lipid families: diacylglycerol, triacylglycerol, hexosyl ceramide, and hydroxy hexosyl ceramide (Figure 1). The relative levels of all other monitored lipid classes were found to differ significantly in fixed and fresh brain tissue, including glycolipids (monoacylglycerol), sphingolipids (ceramide, hydroxy ceramide, phytoceramide, dihydroceramide, sphingomyelin, and sulfatide), and glycerophospholipid classes (phosphatidylcholine, lysophosphatidylcholine, choline plasmalogen, phosphatidylethanolamine, ethanolamine plasmalogens, phosphatidic acid, and phosphatidylserine) and *N*-acylethanolamide.

Diacylglycerols (DAG) were monitored in negative electrospray ionization (NESI) mode using DAG 36:2 [C3] as a stable isotope internal standard. DAG species were observed to be in similar concentrations and rank order as a previous study of human frontal cortical gray matter reported out of our lab (Wood et al., 2021), suggesting consistency in the monitoring of this lipid family. Of 13 monitored DAG species (Supplementary File 1), we found the relative levels of four to be insignificantly different in fixed and fresh human cortical gray matter (Figure 1A). Our results contradict a prior report of as much as a 900% increase in DAG levels of human temporal gyrus fixed *via* immersion (Gaudin et al., 2014).

Triacylglycerols (TAG) were monitored in positive electrospray ionization (PESI) mode using DAG 36:2 [C3] as a stable isotope internal standard. Of eight monitored TAG species (Supplementary File) 1, we identified three with insignificantly different relative levels between fixed and fresh brain tissue samples (Figure 1B). Similar to the DAG results, these TAG species were found to contain unsaturated acyl chains, suggesting this composition may be more structurally resilient to degradation during fixation than their saturated counterparts.

Out of 13 monitored hexosyl ceramide (HexCer) species (Supplementary File 1), we found the relative levels of three to be insignificantly different between fixed and fresh tissue (Figure 1C), while three (of ten) hydroxy hexosyl ceramides (HexCerOH) were found to have insignificant differences (Figure 1D and Supplementary File 1). Both HexCer and HexCerOH were monitored in NESI mode using Cer 16:0 [C16] as a stable isotope internal standard. Relative concentrations of HexCer species were found to be in the same rank order (HexCer 18:0 > HexCer 24:0 > HexCer 20:0) as a prior study of human brain tissue performed in our lab (Wood et al., 2021), indicating reliability in the monitoring of this lipid family. Chloride adducts were used in the monitoring of HexCer and HexCerOH as this method has been shown to provide the greatest sensitivity in the monitoring of ceramide families (Wood and Woltjer, 2021; Wood et al., 2021). However, as has been reported previously, the flow infusion analysis methodology was not able to distinguish between galactosyl- and glucosyl-ceramide classes of HexCer (Wood et al., 2021).

### Survey Study

A broader survey study of fixed brains provided baseline lipid levels of diacylglycerols (DAG), triacylglycerols (TAG), hexosyl ceramides (HexCer), and hydroxy hexosyl ceramides (HexCerOH) across six brain regions: cerebellar cortical gray matter, white matter of the superior cerebellar peduncle, gray and subcortical white matter of the precentral gyrus, posterior limb of internal capsule, and periventricular white matter.

Both DAGs and TAGs were found to be approximately equal between gray and white matter regions in fixed brains (Tables 1, 2). In contrast, DAGs have been shown to be in higher concentrations in fresh white matter versus gray matter (Wood et al., 2021). This indicates some degree of DAG degradation in white matter may have occurred, suggesting DAG stability during systemic fixation may be restricted to gray matter regions. Concentrations of DAG 36:1 were highest in all brain regions, reflecting prior lipidomic investigations of fresh frontal cortex (Wood et al., 2015a,^2021^). TAGs were observed to be in far lower quantities than DAGs, aligning with fresh tissue findings (Wood et al., 2015a). HexCer and HexCerOH were in greater concentrations in white matter regions than gray matter, aligning with findings in fresh tissue (Tables 3, 4; Wood et al., 2021).

**TABLE 1:** Levels of diacylglycerol (DAG) in regions of the formalin fixed human brain (*n* = 21): cortical gray matter (GM) of the cerebellum, white matter (WM) of the superior cerebellar peduncle, cortical gray and subcortical white matter of the precentral gyrus, internal capsule (IC) and periventricular (PV) white matter.

Lipid	Cerebellum		Precentral		IC	PV
DAG	WM	GM	WM	GM	WM	WM
34:0	4.78 ± 1.35	6.31 ± 1.28	5.20 ± 0.97	4.95 ± 0.90	6.24 ± 1.27	4.07 ± 1.01
36:1	27.41 ± 3.27	23.34 ± 2.80	26.74 ± 2.69	24.13 ± 3.41	33.71 ± 3.95	28.39 ± 2.42
36:2	9.11 ± 1.09	10.46 ± 2.01	8.52 ± 0.92	8.30 ± 1.08	10.06 ± 1.27	8.75 ± 0.92
40:4	1.43 ± 0.19	1.82 ± 0.31	1.81 ± 0.16	1.61 ± 0.26	1.58 ± 0.18	11.30 ± 2.71
40:5	0.64 ± 0.08	1.17 ± 0.21	0.87 ± 0.09	0.91 ± 0.14	0.66 ± 0.10	0.57 ± 0.07
40:6	2.81 ± 0.34	4.41 ± 0.68	4.26 ± 0.46	4.12 ± 0.50	2.80 ± 0.38	2.02 ± 0.21

Data are presented as mean R values ± standard error of the mean. Relative levels were calculated as a ratio of the peak area of the endogenous lipid to the peak area of the assigned internal standard, and were subsequently corrected for the wet weight of the sample to obtain the R value (relative level*(10/wet weight [mg])).

**TABLE 2:** Levels of triacylglycerol (TAG) in regions of the formalin fixed human brain (*n* = 21): cortical gray matter (GM) of the cerebellum, white matter (WM) of the superior cerebellar peduncle, cortical gray and subcortical white matter of the precentral gyrus, internal capsule (IC) and periventricular (PV) white matter.

Lipid	Cerebellum		Precentral		IC	PV
TAG	WM	GM	WM	GM	WM	WM
50:1	0.39 ± 0.07	0.28 ± 0.05	0.22 ± 0.07	0.18 ± 0.04	0.24 ± 0.07	0.43 ± 0.14
50:2	0.65 ± 0.09	0.48 ± 0.09	0.37 ± 0.10	0.29 ± 0.06	0.55 ± 0.15	0.61 ± 0.18
52:2	1.39 ± 0.20	0.98 ± 0.18	0.77 ± 0.20	0.62 ± 0.11	1.17 ± 0.33	1.32 ± 0.37
52:3	0.83 ± 0.11	0.64 ± 0.13	0.51 ± 0.04	0.43 ± 0.08	0.72 ± 0.21	0.81 ± 0.24

Data are presented as mean Rvalues ± standard error of the mean. Relativelevels werecalculated as a ratio of the peak area of the endogenous lipid to the peak area of the assigned internal standard, and were subsequently corrected for the wet weight of the sample to obtain the R value (relative level*(10/wet weight [mg])).

**TABLE 3:** Levels of hexosyl ceramide (HexCer) in regions of the formalin fixed human brain (*n* = 21): cortical gray matter (GM) of the cerebellum, white matter (WM) of the superior cerebellar peduncle, cortical gray and subcortical white matter of the precentral gyrus, internal capsule (IC) and periventricular (PV) white matter.

Lipid	Cerebellum		Precentral		IC	PV
HexCer	WM	GM	WM	GM	WM	WM
d18:1/18:0	8.52 ± 0.85	1.17 ± 0.16	4.47 ± 0.37	0.76 ± 0.08	8.85 ± 0.50	6.75 ± 0.34
d18:1/18:1	0.50 ± 0.05	0.05 ± 0.01	0.25 ± 0.04	0.04 ± 0.005	0.42 ± 0.04	0.33 ± 0.03
d18:1/20:0	0.45 ± 0.05	0.09 ± 0.01	0.28 ± 0.03	0.08 ± 0.01	0.44 ± 0.05	0.39 ± 0.04
d18:1/22:1	0.32 ± 0.02	0.05 ± 0.01	0.26 ± 0.03	0.05 ± 0.003	0.37 ± 0.02	0.31 ± 0.02
d18:1/24:0	2.86 ± 0.18	0.52 ± 0.06	2.34 ± 0.17	0.38 ± 0.03	4.05 ± 0.20	4.37 ± 0.27
d18:1/26:1	1.73 ± 0.18	0.31 ± 0.05	1.64 ± 0.19	0.29 ± 0.04	1.96 ± 0.26	2.15 ± 0.28

Data are presented as mean R values ± standard error of the mean. Relative levels were calculated as a ratio of the peak area of the endogenous lipid to the peak area of the assigned internal standard, and were subsequently corrected for the wet weight of the sample to obtain the R value (relative level*(10/wet weight [mg])).

**TABLE 4:** Levels of hydroxy hexosyl ceramide (HexCerOH) in regions of the formalin fixed human brain (*n* = 21): cortical gray matter (GM) of the cerebellum, white matter (WM) of the superior cerebellar peduncle, cortical gray and subcortical white matter of the precentral gyrus, internal capsule (IC) and periventricular (PV) white matter.

Lipid	Cerebellum		Precentral		IC	PV
HexCerOH	WM	GM	WM	GM	WM	WM
d18:1/18:0	1.11 ± 0.10	0.18 ± 0.02	0.53 ± 0.06	0.13 ± 0.01	1.02 ± 0.08	0.73 ± 0.05
d18:1/22:0	2.16 ± 0.22	0.49 ± 0.08	1.81 ± 0.21	0.44 ± 0.07	2.21 ± 0.32	2.13 ± 0.26
d18:1/24:0	9.01 ± 0.54	1.84 ± 0.29	7.67 ± 0.57	1.44 ± 0.18	10.04 ± 0.94	9.67 ± 0.96
d18:1/26:1	1.44 ± 0.13	0.40 ± 0.04	1.56 ± 0.17	0.52 ± 0.08	1.59 ± 20	1.79 ± 0.18

Data are presented as mean R values ± standard error of the mean. Relative levels were calculated as a ratio of the peak area of the endogenous lipid to the peak area of the assigned internal standard, and were subsequently corrected for the wet weight of the sample to obtain the R value (relative level*(10/wet weight [mg])).

## Discussion

This study identified specific species of (DAGs), triacylglycerols (TAGs), hexosyl ceramides (HexCer), and hydroxy hexosyl ceramides (HexCerOH) as having commensurate concentrations in fresh and fixed human cortical gray matter (Figure 1). A follow-up survey study provided baseline levels of such species across six brain regions sampled from fixed, whole body donors. Unexpectedly, sphingomyelin, phosphatidylcholine, plasmalogens, and sulfatides, which have been shown to be unaltered in fixed brain tissue (Heslinga and Deierkauf, 1962; Carter et al., 2011; Harris et al., 2020), were not found to be commensurate between our fresh and fixed samples. This is perhaps due to a component of either the embalming fluid or wetting solution influencing the chemical structure of endogenous lipids. Further, since the fixed brains were removed up to a year prior to sampling, oxidation of cortical lipids at the air-tissue interface is plausible as this brain region was exposed to ambient oxygen following removal (Wolf and Quinn, 2008; Hackett et al., 2011). Conversely, our demonstrated stability of DAG species in embalmed brains contradicts prior findings in human brain specimens fixed *via* immersion, which exhibited substantially elevated levels of these lipids (Gaudin et al., 2014). Importantly, tissue fixation *via* immersion in formaldehyde leads to a gradient of fixation that progresses from superficial to deep (Bauer et al., 2016), leaving deeper tissues vulnerable to autolytic degradation at the cellular level (Garman, 1990; McFadden et al., 2019). It is possible that such degradation could lead to an alteration in the lipid profile, accounting for this discrepancy between tissue that is preserved *via* immersion versus systemic fixation.

**FIGURE 2:**
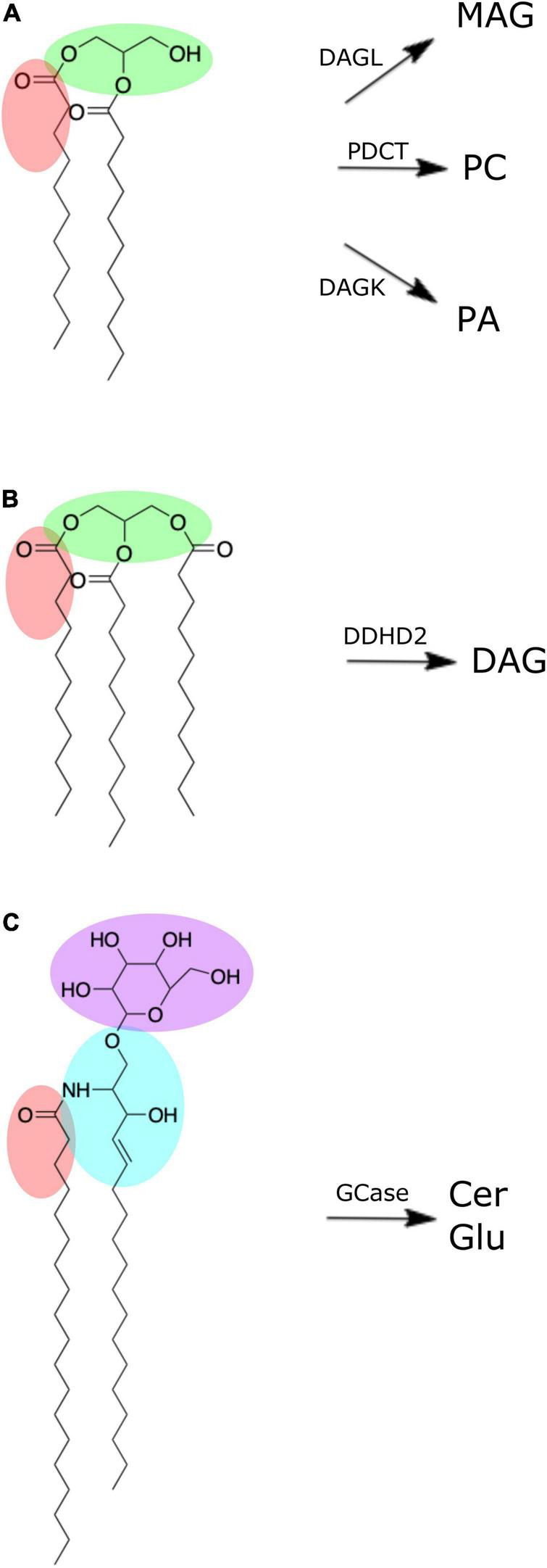
Chemical structures of brain lipid molecules (created using ChemDraw Direct, Version 19.0.0). **(A)** Diacylglycerol is composed of a glycerol molecule (green) bonded to two fatty acid chains (one of which is highlighted red). Diacylglycerol can be metabolized by diacylglycerol lipase (DAGL) to form monoacylglycerol (MAG), phosphatidylcholine diacylglycerol phosphotransferase (PDCT) to form phosphatidylcholine, or diacylglycerol kinase (DAGK) to form phosphatidic acid. **(B)** Triacylglycerol is composed of a glycerol molecule (green) bonded to three fatty acid chains (one of which is highlighted red). Triacylglycerol can be metabolized by DDHD domain containing 2 (DDHD2) to form diacylglycerol. **(C)** Hexosyl ceramide is composed of a ceramide with sphingosine backbone (blue) and fatty acid (red) bonded to either a glucosyl or galactosyl group. If it is a glucosyl group (purple) it forms glucosylceramide, which can be metabolized by β-glucocerebrosidase (GCase) to form ceramide (Cer) and glucose (Glu).

DAGs and TAGs are neutral lipids structurally characterized by a glycerol molecule covalently bonded to two or three fatty acid chains, respectively (Figures 2A,B; Eichmann and Lass, 2015). In the brain, DAGs serve as important structural components of cellular membranes and organelles, and moderators of signal transduction pathways *via* enzyme activation (e.g., protein kinase C) (Mérida et al., 2019). Further, DAG serves as a metabolic precursor to monoacylglycerol *via* DAG lipase, phosphatidic acid *via* DAG kinase, and glycerophospholipids, such as phosphatidylcholine (-ethanolamine) *via* diacylglycerol choline (ethanolamine) phosphotransferase (Topham, 2006; Figure 2A). Such products of DAG metabolism have structural and signaling functions of their own, serving as reservoirs for secondary messengers and mediators of ion transport (Farooqui et al., 2000; Su et al., 2019).

Tight regulation of DAG metabolism is thus essential for healthy cellular function (Carrasco and Mérida, 2007). Breakdown of these regulator mechanisms has therefore unsurprisingly been implicated in the onset and progression of neurological diseases. Elevated levels of DAG have been identified in the frontal cortex of patients with mild cognitive impairment (Wood et al., 2015a,b), suggesting DAG may have biochemical involvement in the pathogenesis of Alzheimer’s disease (Wood et al., 2015b). Further, altered DAG levels have potential as a biomarker for stratifying mild cognitive impairment severity (Wood et al., 2016). Increased concentrations of DAG have also been reported in frontal and visual cortical regions of Parkinson’s disease subjects (Cheng et al., 2011; Wood et al., 2018), and frontal cortex of Lewy body disease subjects (Wood et al., 2018). Our demonstrated stability of DAG in formalin fixation indicate whole body donors may have utility in supporting the expansion of DAG studies on the human brain in these contexts.

Although present in low quantities in the brain (Table 1; Wood et al., 2015a), brain pools of TAG can be hydrolyzed to form DAG *via* a TAG lipase known as DDHD domain containing 2 (DDHD2) (Eichmann and Lass, 2015; Figure 2B). DDHD2 is one of three serine hydrolases, with the DDHD domain named after its four amino acid residues: three aspartates (D) and one histidine (H) (Inoue et al., 2012). Mutations of DDHD2 have been linked to the genetic disorder known as complex hereditary spastic paraplegia (CHSP), leading to tonic spasms of the lower limbs and intellectual disability (Schuurs-Hoeijmakers et al., 2012; Eichmann and Lass, 2015). Capitulating CHSP in mice *via* DDHD2 inhibition led to accumulation of TAG in the brain, primarily in the form of intraneuronal large lipid droplets (Inloes et al., 2014). The stability of TAG in fixed brains suggests these specimens may be useful in supporting investigations into the relationship of DDHD2 and TAG in the context of CHSP.

HexCers (a.k.a. cerebrosides) are glycosphingolipids characterized by either glucosyl or galactosyl moieties linked to the terminal hydroxy group of a ceramide molecule, yielding glucosyl- or galactosylceramide, respectively (Figure 2C). Hydroxylation of the fatty acid produces HexCerOH (Graf et al., 2002). While flow infusion analysis is not able to distinguish between (hydroxy-) glucosylceramide (GluCer) and galactosylceramide (GalCer) (Wood et al., 2021), these lipids collectively serve as major structural components and signaling molecules in neuronal membranes, with understanding of their influence on neuropathology continually expanding (Graf et al., 2002; Reza et al., 2021). Mutation in the gene (*GBA1)* encoding for β-glucocerebrosidase (GCase), the lysosomal enzyme responsible for cleaving GluCer into glucose and ceramide (Figure 2C), leads to elevated levels of GluCer and is the underlying cause of the most common lysosomal storage disorder known as Gaucher’s disease (Vitner et al., 2010; Farfel-Becker et al., 2014). *GBA1* mutations are also the most common genetic risk factor for Parkinson’s disease (Gegg and Schapira, 2018). Decreased GCase activity has been reported in the substantia nigra, cerebellum, putamen, amygdala and anterior cingulate cortex of Parkinson’s disease subjects (Gegg et al., 2012; Murphy et al., 2014) and has been linked to α-synuclein aggregation that characterizes Parkinson’s disease pathogenesis (Murphy et al., 2014). However, despite this altered GCase function, the cerebellum, putamen, and primary motor cortex of Parkinson’s disease subjects did not exhibit any increase in GluCer levels (Clark et al., 2015; Gegg et al., 2015). Clearly, more research is needed to fully elucidate the influence of GCase activity and GluCer levels in the brain, with our results indicating whole body donors may be a suitable model for such pursuits.

The galactosylceramide (GalCer) constituent of HexCers comprises over 20% of the lipid content of the myelin sheath, making it the most abundant single component of this neuron insulator (Norton and Cammer, 1984; Reza et al., 2021), perhaps accounting for elevated proportions of HexCer observed in white matter regions (Table 3). In the central nervous system, the myelin sheath is formed from membranous extensions of oligodendrocytes that coil around and insulate axons in a process called myelination. The coiling of the myelin sheath is dependent on localizations of GalCer (and its sulfated form, galactosylceramide-sulfate) facilitating carbohydrate-carbohydrate interactions between opposing membranous layers (Boggs, 2014). Such interactions are termed “glycosynapses” which function to direct intracellular signaling that dissolves the cytoskeleton, promoting cytosolic flexibility during coiling (Boggs et al., 2008). GalCer is therefore important in the formation, stability, and permeability of myelin (Dyer and Benjamins, 1991; Bosio et al., 1998; Poitelon et al., 2020). Unsurprisingly, GalCer has been implicated in the pathogenesis of demyelinating diseases, including multiple sclerosis and Krabbe’s disease (Blewett, 2008; Giussani et al., 2021). Understanding the biochemical keystones of demyelination is a growing area of research that could theoretically be supported with the employment of fixed brain tissue.

Conserving the teaching utility of brain specimens was a priority for this study and influenced the selection of regions to be investigated. Subcortical structures that would have required destructive dissection were therefore excluded. Beyond its seamless accessibility, the frontal cortical gray matter was chosen for the comparative analysis due to its frequent use in lipidomic analyses of fresh human brain tissue, an indication of this region’s demand as an experimental model. Such studies have revealed altered lipid metabolism in the frontal cortex of subjects with mild cognitive impairment (Wood et al., 2015a,b), Parkinson’s disease (Wood et al., 2018), Lewy body disease (Wood et al., 2018), and Alzheimer’s disease (Wood et al., 2015b), as previously discussed. Regarding the survey study, the cerebellum, which functions to modulate coordinated, fine motor control, was selected because this region has been used in lipidomic studies as a sort of control since it is largely unaltered in neurodegenerative conditions such as Alzheimer’s disease (Couttas et al., 2016). The internal capsule is a concentration of white matter composed of myelinated afferent and efferent projection fibers, connecting the cortex with lower regions of the central nervous system. While altered properties of the internal capsule have been described in idiopathic demyelinating disease like multiple sclerosis (Lee et al., 2000) and neuromyelitis optica spectra disorder (Long et al., 2017), lipidomic studies of this region are currently lacking. The periventricular white matter is a ribbon of myelinated fibers located immediately adjacent to the lateral ventricle. Lesions of the periventricular white matter identified *via* magnetic resonance imaging have been associated with altered memory and executive functioning (Bolandzadeh et al., 2012), however, the lipid profile of such cases is under-studied. These subcortical regions were thoughtfully exposed and sampled in a way that did not detract from the brain’s value as a teaching tool.

The limitations of this study are primarily derived from its limited sample size. Expanding future studies would not only strengthen results, but would allow for the control of more variables, including subject age, sex, and medical history. Follow-up experiments could restrict confounding variables by investigating pre- and post-embalmed brain samples from the same individual. Investigating the influence of the length of storage could also be of great benefit; establishing a timeline of lipid profiles in formalin fixed nervous tissue would not only better elucidate the utility of embalmed brains in metabolic studies, but could also inspire lipidomic investigations of rare, formalin-fixed neuropathological specimens found in historical collections.

## Materials and Methods

### Ethical Approval

The study was conducted in accordance with the Declaration of Helsinki, and the protocol was reviewed and approved by the LMU Institutional Review Board (Ref.# 998V.0).

### Fresh Brain Tissue

Fresh human cortical gray matter used in the comparative analysis was sourced from the Oregon Brain Bank. Tissue was obtained *via* autopsy of individuals who voluntarily donated their tissue after having provided informed consent. Neuropathologists at the Oregon Health Sciences University performed neurological evaluation to ensure subjects used in this study were free of neurological disease. Samples were dissected by the neuropathologist (RLW) before being flash frozen and stored at −80°C. ∼50 mg samples were dissected at the time of analysis and placed in test tubes containing 1 mL of methanol. The demographic information for the individuals who donated the fresh tissue used for the comparative analysis (*n* = 7) is summarized in Table 5.

**TABLE 5:** Demographic information for subjects used in this study.

Parameter	Fresh	Fixed	Fixed
Study	Comparative analysis	Comparative analysis	Survey study
*n*	7	7	21
Age (Yrs ± SD) [Range]	85 ± 11.71 [68–97]	80.28 ± 7.41 [70–92]	74 ± 11.83 [54–94]
PMI (Hrs/Yrs ± SD) [Range]	26.91 ± 25.97 [7–74]	1.79 ± 0.41 [0.89–2.72]	2.03 ± 0.56 [0.92–2.34]
Sex (M:F)	4:3	4:3	15:6

*Hrs, hours; F, female; M, male; PMI, postmortem interval; SD, standard deviation; Yrs, years.*

### Fixed Brain Tissue

Fixed tissue utilized in the comparative analysis and survey study was ethically willed to Lincoln Memorial University DeBusk College of Osteopathic Medicine (LMU-DCOM), with consent to be used in research provided by the individual prior to donating. Donors with a neurodegenerative cause of death listed on their official death certificate or who did not provide consent to be used in research were excluded. Demographic information for donors included in the comparative analysis (*n* = 7) is the survey study (*n* = 21) are included in Table 5.

Individuals were systemically fixed within 48 h of the time of death by trained technicians. Systemic fixation was achieved following the drainage of native blood products *via* introduction of approximately six gallons of embalming fluid (tap water and Carolina’s Perfect Solution [2:1]) through the carotid and/or femoral arteries. Carolina’s Perfect Solution consists of an unpublished ratio of ethanol, aldehyde, alcohol, and 8.5% phenol (Carolina Biological Supply Company, Burlington, NC).

The cranial vault was accessed *via* a circumferential cut of the calvarium through the frontal, occipital and temporal bones. Severance of the dura mater, cranial nerves I-XII, internal carotid and vertebral arteries, and cervical spinal cord allowed the cerebrum, cerebellum, and brainstem to be removed *en bloc*. This procedure occurred no more than 1 year prior to sampling. In the interim, brains were wetted with solution to maintain tissue integrity and prevent microbial growth. This wetting solution was prepared in-house by mixing the following materials: 470 ml of 70% isopropyl alcohol, 1,000 ml mixture of (200 ml Infutrace + 800 ml distilled water), 500 ml 29.5% phenol, 500 ml 10% formalin, and 17.5 L of cold tap water.

**FIGURE 3:**
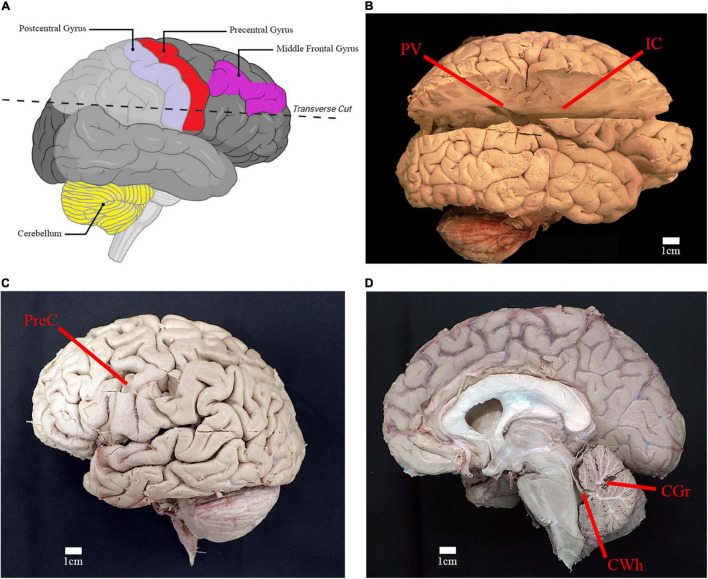
Location of relevant anatomical structures and sites of sampling. **(A)** Illustration of right cerebral hemisphere and location of incision to access deep white matter structures (created using Biorender.com). **(B)** Lateral view of right cerebral hemisphere with incision from parietooccipital sulcus to frontal pole. Location of sampling for periventricular white matter (PV) and posterior limb of internal capsule (IC) are shown. **(C)** Lateral view of left cerebral hemisphere depicting the sampling location for gray matter and subcortical white matter of the precentral gyrus of the frontal lobe (PreC). **(D)** Midsagittal view of right cerebral and cerebellar hemispheres. Sampling location of cerebellar gray matter (CGr) and white matter of the superior cerebellar peduncle (CWh) are shown.

Brains were dissected and sampled by the anatomist (AWB). For the comparative analysis, samples were taken of the cortical gray matter in the region of the middle frontal gyrus (Brodmann area 9) (Figure 3A). For the survey study, samples were taken from six brain regions: gray and subcortical white matter of the left precentral gyrus (primary motor cortex; Brodmann area 4), cortical gray matter of right cerebellar hemisphere, white matter of the superior cerebellar peduncle, right posterior limb of internal capsule, and right periventricular white matter located near the atrium of lateral ventricle (Figures 3B–D). Target sample weight was ∼50 mg, with samples individually placed in test tubes containing 1 mL of methanol.

### Lipid Extraction and Analysis

100 μL of stable isotope internal standard and 1 mL of distilled H_2_O were added to each test tube prior to sample dismemberment *via* sonication. The internal standard contained [16C13] ceramide d18:1/16:0 (3 nmoles), [3C13] DAG 36:2 (1.5 nmoles), [31H2] PE 34:1 (10 nmoles), and [31H2] PC 34:1 (8 nmoles). Following the addition of 2 mL of methyl-tert-butyl ether, samples were shaken and centrifuged (4,000 × g) at room temperature, each for 30 min, separating samples into upper organic and lower aqueous layers. 1 mL of the organic layer was added to 96-well plates, which were dried overnight *via* centrifugal vacuum evaporation. Each sample was introduced to 200 μL of infusion solvent (160 mL 2 propanol, 80 mL methanol, 40 mL chloroform, 1 mL H_2_0, 75 mg NH_4_Cl) before centrifugation of plates (4,000 × g) at room temperature for 15 min to precipitate any particulates (Wood, 2017).

Brain lipids were analyzed *via* high-resolution (140,000; < 3 ppm mass error; at m/z 200-1200) constant infusion electrospray ionization (ESI) (10 μL/min) using an orbitrap mass spectrometer (Thermo Q Exactive). Samples were scanned in both positive-ESI and negative-ESI for.5 min each. Between each sample injection, syringe and infusion line were cleaned with methanol and hexane wash (hexane, ethyl acetate, and chloroform [3:2:2]), respectively, to limit memory effects (Wood, 2017).

### Statistical Analysis

The identification of monitored lipids was based on calculated masses obtained from the Lipid Maps structure database (lipidmaps.org). Lipids found to be within 3 ppm mass error of validated masses were selected for analysis. Relative levels were calculated as a ratio of the peak area of the endogenous lipid to the peak area of the assigned internal standard, and were subsequently corrected for the wet weight of the sample to obtain the *R* value [relative level*(100/wet weight[mg])]. To ascertain the spread of the data and facilitate subsequent calculations, descriptive statistics [mean, standard deviation (SD), and standard error of the mean (SEM)] were performed on the *R* values for each lipid species. Lipids with a relative standard deviation (RSD) > 100 were excluded (RSD = SD/mean)*100. For the comparative analysis, mean *R* values for each lipid species were compared between fresh and fixed samples using two-sample *t*-tests (*p* < 0.05). All statistical analyses were performed in Microsoft Excel (Version 16.50).

## Conclusion

The lipid profiles of fresh and formalin fixed cortical gray matter of whole body donors were compared. Species of diacylglycerols, triacylglycerols, hexosyl ceramides, and hydroxy hexosyl ceramides were found to be insignificantly different between the two groups, indicating fixed brains may be suitable for the analysis of these lipid species. A survey study of 21 donors provided baseline levels of these lipids across white and gray matter brain regions: cerebellar cortical gray matter, white matter of the superior cerebellar peduncle, cortical gray and subcortical white matter of the precentral gyrus, periventricular white matter, and white matter of the posterior limb of the internal capsule.

## Data Availability Statement

The raw data supporting the conclusions of this article will be made available by the authors, without undue reservation.

## Ethics Statement

The studies involving human participants were reviewed and approved by the Lincoln Memorial University Institutional Review Board (Reference #998V.0). The patients/participants provided their written informed consent to participate in this study.

## Author Contributions

AB, KH, BD, RW, and PW conceptualized the project and conducted review and editing. AB, KH, and PW carried out methodology and conducted data analysis. RW provided fresh brain tissue. AB wrote the original draft. BD and PW supervised the project. PW secured resources and funding for lipid analysis. All authors wrote the manuscript and have given approval to the final version of the manuscript.

## Conflict of Interest

The authors declare that the research was conducted in the absence of any commercial or financial relationships that could be construed as a potential conflict of interest.

## Publisher’s Note

All claims expressed in this article are solely those of the authors and do not necessarily represent those of their affiliated organizations, or those of the publisher, the editors and the reviewers. Any product that may be evaluated in this article, or claim that may be made by its manufacturer, is not guaranteed or endorsed by the publisher.
